# Social Experience Regulates Endocannabinoids Modulation of Zebrafish Motor Behaviors

**DOI:** 10.3389/fnbeh.2021.668589

**Published:** 2021-05-11

**Authors:** Stephen A. Orr, Sungwoo Ahn, Choongseok Park, Thomas H. Miller, Miki Kassai, Fadi A. Issa

**Affiliations:** ^1^Department of Biology, East Carolina University, Greenville, NC, United States; ^2^Department of Mathematics, East Carolina University, Greenville, NC, United States; ^3^Department of Mathematics, North Carolina A&T State University, Greensboro, NC, United States; ^4^Department of Biochemistry and Molecular Biology, Brody School of Medicine, East Carolina University, Greenville, NC, United States

**Keywords:** social experience, aggression, zebrafish, Mauthner cell, endocannabinoid, 2-AG, motor circuits

## Abstract

Social status-dependent modulation of neural circuits has been investigated extensively in vertebrate and invertebrate systems. However, the effects of social status on neuromodulatory systems that drive motor activity are poorly understood. Zebrafish form a stable social relationship that consists of socially dominant and subordinate animals. The locomotor behavior patterns differ according to their social ranks. The sensitivity of the Mauthner startle escape response in subordinates increases compared to dominants while dominants increase their swimming frequency compared to subordinates. Here, we investigated the role of the endocannabinoid system (ECS) in mediating these differences in motor activities. We show that brain gene expression of key ECS protein pathways are socially regulated. Diacylglycerol lipase (DAGL) expression significantly increased in dominants and significantly decreased in subordinates relative to controls. Moreover, brain gene expression of the cannabinoid 1 receptor (CB_1_R) was significantly increased in subordinates relative to controls. Secondly, increasing ECS activity with JZL184 reversed swimming activity patterns in dominant and subordinate animals. JZL184 did not affect the sensitivity of the startle escape response in dominants while it was significantly reduced in subordinates. Thirdly, blockage of CB_1_R function with AM-251 had no effect on dominants startle escape response sensitivity, but startle sensitivity was significantly reduced in subordinates. Additionally, AM-251 did not affect swimming activities in either social phenotypes. Fourthly, we demonstrate that the effects of ECS modulation of the startle escape circuit is mediated via the dopaminergic system specifically via the dopamine D1 receptor. Finally, our empirical results complemented with neurocomputational modeling suggest that social status influences the ECS to regulate the balance in synaptic strength between excitatory and inhibitory inputs to control the excitability of motor behaviors. Collectively, this study provides new insights of how social factors impact nervous system function to reconfigure the synergistic interactions of neuromodulatory pathways to optimize motor output.

## Introduction

Social status can be defined by a set of behaviors that accompanies an animal’s position in a social hierarchy. Aggressive behavior typically displayed by dominant animals consists of either physical attacks or pursuit of conspecifics. When two adult male zebrafish are paired in a tank, they quickly establish a stable social relationship in which one fish is dominant and the other is subordinate. These social relationships can be used as the basis to study the effects of social status on behavior and brain function (Miller et al., 2017; Clements et al., 2018; Park et al., 2018).

**FIGURE 1:**
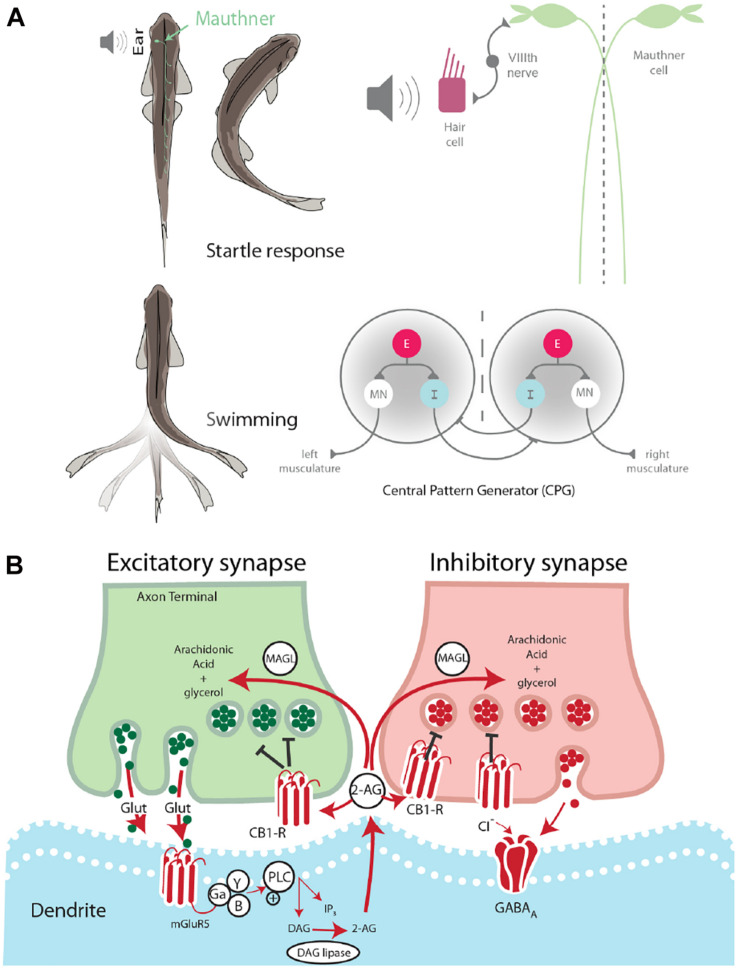
Startle escape and swim motor behaviors are socially regulated. **(A)** Startle behavior in zebrafish is controlled by the M-cell startle escape circuit. The auditory startle escape response is activated when a sound activates hair cells within the ear. Next, the signal is sent from the VIIIth nerve to the M-cell, which activates contralateral fast motor neurons responsible for contraction of flexor muscles that leads to the startle escape response. The swimming motor pattern is controlled by the central pattern generators (CPGs) which repeat along the length of the spinal cord. Each half-center of the CPG is composed of an excitatory interneuron (E), an inhibitory interneuron (I), and a motor neuron (MN). The motor neurons project ipsilaterally to the trunk musculature and induce contraction. **(B)** Schematic model of endocannabinoid retrograde signaling. The endocannabinoid 2-AG is synthesized post-synaptically in response to neurotransmitter binding. Traditional model suggests that retrograde transmission of 2-AG inhibits further release from both excitatory and inhibitory neurons. DAG lipase synthesizes 2-AG post-synaptically. CB1 receptor binds 2-AG. MAGL degrades 2-AG in presynaptic terminal.

Two fundamental behaviors in zebrafish, startle escape and swimming, are notable for the relative simplicity of the neural circuits that control them and the ease with which they can be studied behaviorally and physiologically. The neural circuits underlying these basic motor behaviors have been well-characterized in terms of their neuronal organization (Eaton et al., 2001) and the neurochemicals that modulate their activation (McLean and Fetcho, 2004). The startle escape response in zebrafish and other teleost fish is controlled by a group of reticulospinal neurons, namely the Mauthner cell (M-cell) and two serial homologs, MiD2cm and MiD3cm (Eaton et al., 2001). The firing of a single M-cell is necessary and sufficient for the initiation of a fast startle escape response. The M-cells act as integration centers for auditory, tactile, and visual inputs, and, as such, they are responsible for the initiation of startle escape behavior in response to auditory stimuli (Eaton et al., 2001). Auditory stimuli activate hair cells in the ear, which signals the M-cell via the VIIIth cranial nerve. A stimulus sufficient to activate the M-cell subsequently activates fast motor neurons (MNs) responsible for startle escape and inactivates slow MNs responsible for rhythmic swimming. This activation pattern generates a contralateral contraction of the trunk musculature producing a fast escape away from the stimulus (Eaton et al., 2001; Figure 1A).

Swimming is a well-conserved behavior whose neural circuit has been described in zebrafish (Fetcho and McLean, 2010; Kiehn, 2011). This behavior is controlled by a distributed network of neurons arranged hierarchically from the midbrain to the spinal cord. Initiation of locomotion begins in the mesencephalic locomotor region. This brain region sends descending inputs to reticulospinal neurons in the hindbrain, which project to the central pattern generators (CPGs). The CPG consists of two half-centers, one on either side of the midline (Figure 1A). Each half-center is composed of motor neurons, descending excitatory interneurons (e-INs), and commissural inhibitory interneurons (i-INs). The coordinated action of these neurons is responsible for the locomotor pattern generation (Roberts et al., 2008). The behavioral switch between startle escape and swimming is controlled by a hardwired neural circuit spanning from the hindbrain to the spinal cord. The threshold for this switch from swimming to startle has been shown to be modulated neurochemically by the endocannabinoid 2-arachidonoylglycerol (2-AG) (Song et al., 2015). It has also been demonstrated in goldfish that the reticulospinal M-cells release 2-AG in order to regulate their own excitability (Cachope et al., 2007).

The ECS is broadly involved in the central nervous system and functions via a retrograde signaling mechanism. This neurochemical system is composed of cannabinoid receptors and their endogenous lipid-based ligands, i.e., endocannabinoids. Two cannabinoid receptors have been identified in vertebrates, the cannabinoid 1 receptor (CB1) and the cannabinoid 2 receptor (CB2). While CB1 is the primary cannabinoid receptor found in the brain, CB2 is also present, although at much lower receptor number per cell and is found primarily on immune cells (Lam et al., 2006). The endogenous ligands anandamide and 2-AG are retrograde signaling molecules, which are synthesized “on demand” in response to post-synaptic depolarization (Kano et al., 2009). The synthesis of 2-AG in the post-synaptic neuron is triggered by intracellular increase in Ca^2+^ concentration resulting from cell depolarization. Binding and activation of presynaptic CB1 leads to the pre-synaptic closing of Ca^2+^ channels and/or opening of K^+^ channels. These cellular changes result in reduced neurotransmitter release (Hernandez and Cheer, 2015). After being transported into the presynaptic neuron by an unknown uptake mechanism (Fu et al., 2011), 2-AG is degraded by monoacylglycerol lipase (MAGL) as a mechanism to regulate 2-AG activity (Dinh et al., 2002).

It has been demonstrated that the endocannabinoid 2-AG acts as a molecular “clutch” in the zebrafish spinal cord circuit, setting the threshold for the switch from swimming to startle escape behavior (Song et al., 2015). Furthermore, evidence strongly suggests that the M-cell releases 2-AG (Cachope et al., 2007). It was found that activation of the group 1 metabotropic glutamate receptor (mGluR1) led to a lasting potentiation from the VIIIth nerve onto the M-cell. 2-AG is known to be synthesized and released from a post-synaptic cell in response to mGluR1 activation. Moreover, blockage of CB1 eliminated this potentiation (Cachope et al., 2007). These results suggest that the M-cell increases its own excitability by releasing 2-AG. The findings from these two studies set the stage to study the role of the ECS in balancing activation of the startle escape and swimming circuits based on social status.

Socially dominant fish show reduced startle escape sensitivity and higher swimming frequency, whereas socially subordinate fish show a shift in circuit activation toward higher sensitivity of the M-cell startle escape and lower activation of the swimming circuit resulting in lower swimming frequency (Miller et al., 2017). While the effects of social status on behavior are well-documented, the effects of social status on the molecular machinery responsible for shifting activation between the competing neural circuits of escape and swim is poorly understood. The known role of the ECS in switching activation between motor circuits suggests potential involvement in the facilitation of social status-dependent shifts in motor behavior (Figure 1B). However, this social role of the ECS remains undetermined. Here, we investigated the effects of ECS modulation on the social status-dependent activation of two competing motor circuits controlling the M-cell startle escape reflex and swimming behaviors.

## Materials and Methods

### Animal Maintenance

Zebrafish (*Danio rerio*) were housed at the Zebrafish Core Facility at East Carolina University. The facility was kept at a temperature of at 28°C under a 14 h/10 h light/dark cycle. Fish were fed daily with a high protein commercial food (Otohime B2, Reed Mariculture, Campbell, CA, United States) and with newly hatched artemia (Brine Shrimp Direct, Ogden, UT, United States). Wildtype (AB) zebrafish were group-housed in 10 gallon mixed-sex tanks prior to isolation and pairing. All experiments were performed in accordance with the Institutional Animal Care and Use Committee at East Carolina University (AUP #D320a). The dopamine type 1 receptor knockout line [drd1b^(–/–)^] was generously provided by the Nicolson’s lab (Oregon Health Sciences University). The line was originally constructed at the Sanger Institute with an AB genetic background (Busch-Nentwich et al., 2013) and later deposited at the Zebrafish International Resource Center (ZIRC).

### Social Isolation and Pairing

Adult male fish (∼6–12 months old) were taken from their communal tanks and isolated in a tank for 1 week, separated spatially and visually from other fish to minimize pre-existing social experience (Miller et al., 2017). Subsequently, two animals of equal size were paired in a new tank for a 2-week period and their aggressive behavior was monitored daily for 5 min to assess dominance as described previously (Miller et al., 2017).

### Experimental Setup

After the pairing phase was completed, fish were temporarily separated, and behavioral testing was performed on a single fish following the protocol described elsewhere (Issa et al., 2011). Each fish was placed in a testing chamber (dimensions: 11 × 4 × 3 cm). A pair of conductive electrodes placed on either side of the chamber recorded the electric field potentials. Bare electrodes were 1 mm in thickness with 3–5 mm metal exposure. Electrodes were connected to an AC differential amplifier (AM-Systems model 1700, Carlsborg, WA, United States), and signals were amplified 1,000-fold. Electrical signals were low-pass filtered at 300 Hz and high-pass filtered at 1 KHz. Electrical field potentials are generated by muscle contractions when the fish moves (Issa et al., 2011). These signals were digitized using a Digidata-1322A digitizer then stored using Axoscope software (Molecular Devices, Inc., Sunnyvale, CA, United States). The experimental animals were acclimatized for 30 min before behavioral testing was initiated. Swimming behavior was recorded immediately following acclimation. Immediately after, startle escape responses were recorded.

### Determination of Startle Escape Sensitivity

Auditory pulses consisting of phasic 4 ms sine waves were generated using Audacity open-source audio editor and recorder software^1^. Sound intensity was measured and calibrated external to the tank using a decibel meter (Sinometer, MS6700). Sensitivity of the animal’s auditory startle escape response was determined by tracking startle escape probability as a function of sound intensity. Activation of the M-cell mediated escape has a short latency of 5–15 ms. Non-Mauthner mediated responses with a time onset ranging from 15 to 40 ms were not counted, as these are controlled by an independent set of neural circuit that is not the target of our investigation (Eaton et al., 2001). Pulse intensity ranged from 70 to 100 dB with 5 dB increments. Pulse intensities were randomized and presented with a minimum of 2-min intervals to prevent habituation of the startle reflex. Response probability for each intensity was tabulated, and these probabilities were averaged across animals.

### Measurement of Swimming Activity

Following the 30-min acclimation period, and before conducting startle escape experiments, the animal’s swimming behavior was recorded for 1 min. The same methods of data acquisition, amplification, digitization, and storage were used as previously stated. Swimming activity was measured by counting swim bursts with Clampfit software. The “Threshold” function was used for this purpose. A potential was marked as a swim burst if it was at least 8 mV in total amplitude and 30–200 ms in duration. This range was chosen based on the typical characteristics of rhythmic swimming potentials that we observed. The timing of each swim burst was saved into a Microsoft Excel spreadsheet in reference to the recording start time.

### Data Analysis

Startle escape and swimming behavioral data was analyzed using Prism (GraphPad software Inc., San Diego, United States) and IBM-SPSS (RRID:SCR_002865). Unless specified otherwise, all comparisons were first subjected to one-way ANOVA or mixed design (a mixture of between-group and repeated-measures variables) ANOVA (between factor as group; within-factors as treatment and decibel) followed by the least significant difference (LSD) or paired two-sided *t*-test *post hoc* test for all multiple comparisons. Before using mixed-design ANOVA, sphericity was tested by using Mauchly’s test. When the assumption of sphericity was violated, the degree of freedom in Greenhouse-Geisser correction was used. For startle escape data, nonlinear regressions were performed using the Boltzmann sigmoidal equation:

Y=Bottom+(Top-Bottom)/(1+exp((V50-X)/Slope)).

### Pharmacology

A day after initial behavioral testing, fish were treated with either AM-251 or JZL184 and re-tested according to the previously stated protocol. Paired fish were separated with a divider during the injection and post-testing phase. The acclimation period was initiated 2 h post-injection. Fish were treated with a drug injected intraperitoneally following the protocol of Song et al. (2015). Intraperitoneal injections are preferred over direct brain injections because there is less risk of altering behavior with the physical injection and because both drugs can effectively cross the blood-brain barrier (Song et al., 2015). The drugs AM-251 and JZL184 were dissolved in DMSO to produce a 40 mM stock solution. For injection, capillary tubing was used, having the dimensions 1.0 mm OD × 0.5 mm ID × 100 mm in length. These were pulled using Flaming/Brown Micropipette Puller – Model P-87 from Sutter Instrument Co. The 40 mM stock solution was diluted in saline to 400 μM AM-251 and 400 μM JZL184. The tip of the micropipette was broken off with a razor blade, before loading with the drug solution. Loaded micropipettes were placed in Pneumatic PicoPump PV 820 for drug administration. A 0.3 % tricaine solution was used to anesthetize the animal prior to injection. Zebrafish were determined to have an average weight of 100 mg, therefore 2 μL of drug was injected to achieve a concentration of 4 mg/kg AM-251 and 4 mg/kg JZL184. To control for injury from injection and possible effects from solvents, separate dominant-subordinate pairs were injected with 10% DMSO in saline. To control for social status, communal fish were injected with either AM-251 or JZL184.

### Molecular Methods

#### ECS Signaling Molecules RNA Extraction and Reverse Transcription

Fish were euthanized by hypothermic shock for 10 min. Dissections were performed in ice cold reverse osmosis water and completed within 5 min of sacrifice. Whole-brain and Hindbrain tissues were collected and stored at −80°C until use. Samples were homogenized by sonication in TRIzol^®^ (Life Technologies) and incubated at room temperature for 5 min. Chloroform was added (200 μL per 1 mL TRIzol^®^), mixed by inversion, and incubated at 4°C for 20 min with intermittent mixing. Samples were then centrifuged at 4°C for 15 min at 14,000 rpm. An equal volume of cold 100% ethanol was added, and sample was passed through a RNeasy^®^ Mini Spin Column (Qiagen) according to RNeasy^®^ protocols. RNA extracts were quantified by Nanodrop 2000 (Thermo Fisher Scientific) and stored at −80°C until use. cDNA synthesis was performed using QuantiTect Reverse Transcription Kit (Qiagen).

#### Qualitative Real-Time PCR

Primers used for the ECS genes are summarized in Table 1. qPCR was performed used the Quantstudio 12k Flex System (Applied Biosystems) to determine expression of dagl, mgl, and cb1r in whole-brain tissue. Beta-actin 2 (actb2) was used an internal reference gene. All samples were run in duplicate. Expression was normalized to actb2 and analyzed using the comparative ΔΔCt method with isolate animals as control.

**TABLE 1:** Primers used for qPCR analysis.

Gene	Forward primer 5′-3′	Forward primer 5′-3′	Amplicon GenBank#	Size (bp)
actb2	CCAAACCCAAGTTCAGCCATGG	TGGATGGGAAGACAGCACGG	NM_181601	118
dagl	CCTGGACACCTCAAATTCGCC	TCCGGTGAGCACAATAGGGA	XM_691781	145
mgl	GGAGACGCCGACAAACTGTG	AGTCGTGATGTAGGGCATGGT	NM_200297	118
cblr	CTCTGGAAGGCCCACCATCAT	CGGATGTCCATGCGTGCC	NM_212820	128

### Western Blot

For western blot analysis four separate western blot trials were conducted with 10 brains per trial for each social phenotype. Zebrafish were anesthetized with 0.02% MS-222 (1 min) then placed in iced water (10 min). Brains were dissected out, placed in a 1.5 mL microcentrifuge tube, and stored at -20°C until use. Then they were prepared using the total membrane isolation protocol. Brains were homogenized in 1 ml of resuspension buffer: 2.5 ml 2M Sucrose, 2 ml 10 × 10 mM Tris–HCl, 400 μL 0.25M EDTA, 40 μl 20x protease cocktail inhibitor. The homogenized brains were centrifuged (2,000 rpm, 4°C, 10 min) followed by ultra-centrifugation (37,000 rpm, 4°C, 60 min). Protein sample concentrations were determined with a Lowery protein assay. For Western blots, 10 μg of each protein sample was denatured using 4X buffer containing 10X reducing reagent at 70°C for 10 min and loaded onto a Mini-PROTEAN^®^ TGX^TM^ Precast Protein Gels (Bio-Rad, Hercules, CA, United States) and run ∼120 min at 60V. The proteins were transferred to a 0.2 μm Nitrocellulose membrane using *Trans-*Blot^®^ Turbo^TM^ RTA Mini Transfer Kit (Bio-Rad, Hercules, CA, United States). The membrane was blocked with PBS containing 5% (w/v) nonfat dry milk and 0.1% (v/v) Tween 20 for 1 h at room temperature and then incubated with primary antibodies overnight at 4°C. After three washes with PBS containing 0.1% (v/v) Tween 20, the membrane was incubated with HRP-conjugated goat anti-mouse or goat anti-rabbit IgG (Cell Signaling) secondary antibody for 1 h. After washing, protein detection was performed using Horseradish peroxidase (HRP) Detection kit (SuperSignal^TM^ West Pico, Thermo Fisher Scientific) and visualized using the ChemiDoc Imaging System (Bio-Rad). The intensity of bands was quantified with ImageLab software. Band intensity was normalized by calculating protein/β-actin ratio. Data from dominants and subordinates were then normalized to protein/β-actin values from communal fish (Bio-Rad, Hercules, CA, United States). Primary antibodies used were as follows: mouse anti-CB_1_R (1:500 Novus Biologicals), Rabbit anti-DAGL (1:500 Bioss Antibodies), β-actin (1:1000 Cell Signaling).

### Neurocomputational Model

We constructed a neurocomputational model network that is composed of one excitatory cell, one inhibitory cell, and one M-cell. In the model, auditory inputs are initially delivered to the excitatory cell, which then excites the M-cell and the inhibitory cell. The inhibitory cell inhibits the M-cell only. In other words, auditory inputs affect the M-cell via two paths: a direct path via the excitatory cell and an indirect path via the excitatory cell and then the inhibitory cell. All model neurons were modeled as a conductance-based modified Morris–Lecar model with additional calcium-dependent potassium current (Morris and Lecar, 1981; Izhikevich, 2007; Ermentrout and Terman, 2010; Miller et al., 2017; Park et al., 2018). The membrane potential of each cell obeys the following current balance equation:

(1) $$C\frac{dv}{dt}=-I_{Ca}-I_{K}-I_{L}-I_{KCa}-I_{Tot}$$

where $I_{K}=g_{K}n\left(v-v_{K}\right),I_{Ca}=g_{Ca}m_{\infty }\left(v\right)\left(v-v_{Ca}\right),I_{KCa}=g_{KCa}\left\{\frac{\left[Ca\right]}{\left[Ca\right]+k_{1}}\right\}\left(v-v_{K}\right)$, *I*_*L*_ = *g*_*L*_(*v*−*v*_*L*_) represent the potassium, calcium, calcium-dependent potassium, and leak currents, respectively. [*Ca*] represents intracellular calcium concentration. For all neurons, *g*_*K*_ = 8, *v*_*K*_ = −84,*g*_*L*_ = 2, *v*_*L*_ = −60,*g*_*Ca*_ = 4, *v*_*Ca*_ = 120, and *k*_1_ = 10. In the M-cell, *g*_*KCa*_ = 0.3 and *C=1*. In other neurons, *g*_*KCa*_ = 0.25 and *C=20*.

*m*_*∞*_ is an instantaneous voltage-dependent gating variable for the calcium current where,

(2) $$m_{\infty }\left(v\right)=0.5\left(1+\tanh\left(\frac{v-v_{1}}{v_{2}}\right)\right)$$

with *v*_1_ = −1.2 and *v*_2_ = 18.

The concentration of intracellular Ca^2 +^ is governed by the calcium balance equation:

(3) $$\frac{d\left[Ca\right]}{dt}=\varepsilon \left(-\mu I_{Ca}-k_{Ca}\left[Ca\right]\right)$$

where ε = 0.005,μ = 0.19 for all neurons. *k*_*Ca*_ = 0.9 in the M-cell and *k*_*Ca*_ = 1 in other neurons.

*n* is a gating variable for the potassium current obeying,

(4) $$\frac{dn}{dt}=\frac{\phi \left(n_{\infty }\left(v\right)-n\right)}{\tau_{n}\left(v\right)}$$

(5) $$n_{\infty }\left(v\right)=0.5\left(1+\tanh\left(\frac{v-v_{3}}{v_{4}}\right)\right)$$

(6) $$\tau_{n}\left(v\right)=1/\text{cosh}\left(\frac{v-v_{3}}{2v_{4}}\right)$$

where ϕ = 0.23, *v*_3_ = 12, and *v*_4_ = 17 for all neurons.

In an excitatory cell and an inhibitory cell, the synaptic variable, *s*, is modeled by an equation for the fraction of activated channels,

(7) $$\frac{ds}{dt}=\alpha s_{\infty }\left(v\right)\left(1-s\right)-\beta s$$

where $s_{\infty }\left(v\right)=1/\left(1+\exp\left(-\frac{v+\theta_{s}}{\sigma_{s}}\right)\right)$ with θ_*s*_ = 0 and σ_*s*_ = 4. The parameters α = 15 and β = 0.3 in an excitatory cell, and α = 8.5 and β = 0.046 in an inhibitory cell.

*I*_*Tot*_ represents the total input that a cell receives and is composed of a fixed constant (*I*_*0*_), the synaptic current (*I*_*syn*_) which represents the sum of synaptic inputs from other cells, and an applied current [*I*_*app*_(*t*)]. The synaptic current is given by:

(8) $$I_{syn}=g_{syn}\left(v-v_{syn}\right)\sum_{j}s_{j}$$

where the summation is over *s* variables from all neurons projecting to a given neuron.

In the current neuronal network, an excitatory cell does not receive any synaptic input but receives an external stimulus to simulate the effect of an external stimulus from the sensory input. Thus, in an excitatory cell, *I*_*Tot*_ = *I*_*E*0_ + *I*_*syn*_ + *I*_*app*_(*t*) where *I*_*E*0_ = 43.9 is a fixed constant, *I*_*syn*_ = 0, and *I*_*app*_(*t*) = *W*_*E*_*I*(τ). Here, *W*_*E*_ is the stimulus strength, and *I*(τ) is the stimulus which resembles the square unit pulse with height 1 with duration of 2 ms starting at time τ.

In the M-cell, which receives synaptic inputs from an excitatory cell and an inhibitory cell in the network, the synaptic input is,

(9) $$I_{syn}=g_{\text{E}\to \text{M}}\left(v-v_{\text{E}\to \text{M}}\right)s_{E}+g_{\text{I}\to \text{M}}\left(v-v_{\text{I}\to \text{M}}\right)s_{I}+g_{\text{M}\to \text{M}}\left(v-v_{\text{M}\to \text{M}}\right)s_{M}$$

where *s*_*M*_ is the synaptic variable from another M-cell, which is assumed to be a constant in the current study.

Now, calcium is known to modulate the presynaptic neurotransmitter release via retrograde signaling (Diana and Bregestovski, 2005). In the model M-cell we assumed that intracellular calcium level reciprocally modulates the presynaptic input to the M-cell and *I*_*syn*_ is updated as follows:

(10) $$I_{syn}=g_{\text{I}}*g_{\text{E}\to \text{M}}*\left(v-v_{\text{E}\to \text{M}}\right)s_{E}+g_{\text{I}}*g_{\text{I}\to \text{M}}*\left(v-v_{\text{I}\to \text{M}}\right)s_{I}+g_{\text{M}\to \text{M}}\left(v-v_{\text{M}\to \text{M}}\right)s_{M}$$

where *g*_*I*_ obeys the following equation:

(11) $$\frac{dg_{I}}{dt}=\frac{\frac{g_{Imax}}{\left[Ca\right]+k_{2}}-g_{1}}{\rho }$$

where *g*_*Imax*_ is the maximal *g*_*I*_ value, ρ is the time constant of *g*_*I*_, and [Ca] is the intracellular calcium concentration of the M-cell. The total input to the M-cell is *I*_*Tot*_ = *I*_*M*0_ + *I*_*syn*_ where *I*_*M*0_ = 31 is a fixed constant and the synaptic input *I*_*syn*_ is as in Eq. (10). Other parameter values are given as follows: *g*_E→M_ = 0.15, *v*_E→M_ = 30, *g*_I→M_ = 0.5, *v*_I→M_ = −50, *g*_M→M_ = 0.5, *v*_M→M_ = −50, *s*_*M*_ = 0.029, *g*_*Imax*_ = 20, *k*_2_ = 10, and ρ = 10000.

An inhibitory cell does not receive any direct external stimulus [*I*_*app*_(*t*) = 0] but receives a synaptic input from the excitatory cell. Thus, in the inhibitory cell, *I*_*syn*_ = *g*_I_*g_E→I_(*v*−*v*_E→I_)*s*_*E*_ where *s*_*E*_ is the synaptic variable from the excitatory cell. Thus, the total input to an inhibitory cell is *I*_*Tot*_ = *I*_*I*0_ + *I*_*syn*_ where *I*_*I*0_ = 36, *v*_E→I_ = 30, and *g*_E→I_ = 0.75 for a dominant-like model and *g*_E→I_ = 0.7 for a subordinate-like model.

Some parameters were modified to reflect different firing properties of each cell based on experiments (Eaton et al., 2001; Korn and Faber, 2005; Liao and Fetcho, 2008; Song et al., 2015). We note that all three cells are excitable cells so that they do not fire action potentials unless they receive enough excitatory inputs from other active cells or external stimulus. The main parameter that we controlled to implement these firing properties is the baseline level of a fixed constant *I*_*0*_. Note that only the excitatory cell will receive the external stimulus which mimics the sensory input.

2-AG released from the M-cell modulates the release of the neurotransmitters in the pre-synaptic cells through CB_1_R. To explore how 2-AG modulates the observed social status dependent escape responses to the external stimulus, we implemented the 2-AG modulation of synaptic inputs in the cells as follows.

In an inhibitory cell,

(12) $$I_{Tot}=I_{I0}+I_{syn}=I_{I0}-g_{\text{I}}*g_{\text{E}\to \text{I}}\left(1+CB1R_{EI}\right)\left(v-v_{\text{E}\to \text{I}}\right)s_{E}$$

The main parameter CB1R_*EI*_ depends on the social status and we let CB1R_*EI*_ = 0.32 for a dominant-like model and CB1R_*EI*_ = 0.3 for a subordinate-like model.

In the M-cell,

(13) $$I_{Tot}=I_{M0}+I_{syn}=I_{M0}-g_{\text{I}}*g_{\text{E}\to \text{M}}\left(1+CB1R_{EM}\right)\left(v-v_{\text{E}\to \text{M}}\right)s_{E}-g_{\text{I}}*g_{\text{I}\to \text{M}}\left(1-CB1R_{IM}\right)\left(v-v_{\text{I}\to \text{M}}\right)s_{I}-g_{\text{M}\to \text{M}}\left(v-v_{\text{M}\to \text{M}}\right)s_{M}$$

The main parameters CB1R_*EM*_ and CB1R_*IM*_ depend on the social status. We let CB1R_*EM*_ = 0.27 for a dominant-like model and 0.3 for a subordinate-like model. Similarly, CB1R_*IM*_ = 0.2 for a dominant-like model and 0.25 for a subordinate-like model.

Simulations were performed on a personal computer using the software XPP (Ermentrout, 2002). The numerical method used was an adaptive-step fourth order Runge-Kutta method with a step size 0.01 ms. The neurocomputational model is available online in ModelDB^2^. This website offers one of the largest opensource selections of neurocomputational models for various brain regions.

## Results

### Social Status Regulation of Endocannabinoids’ Signaling Pathways

**FIGURE 2:**
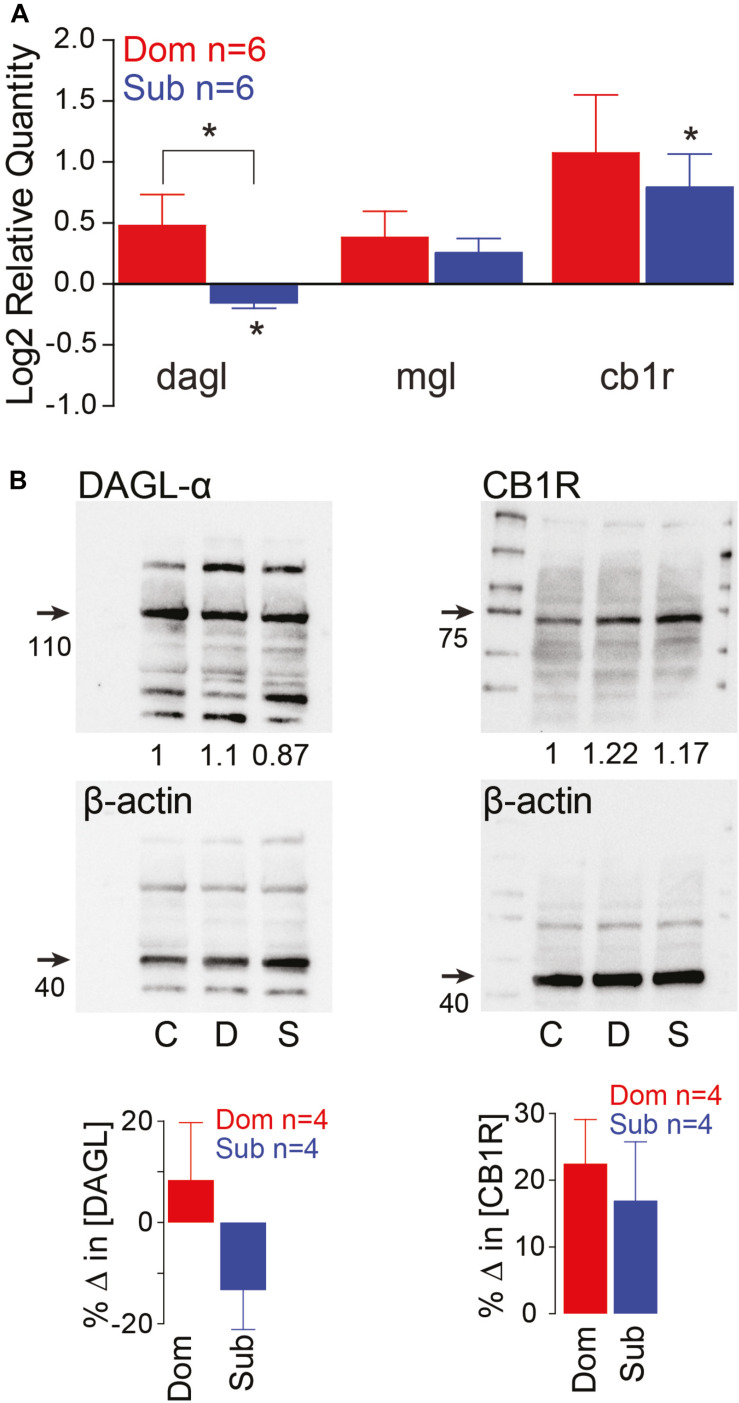
Endocannabinoid signaling pathway is socially regulated and its modulation of M-cell excitability is status-dependent. **(A)** qPCR gene expression analysis of ECS signaling molecules. Data of dominants and subordinates normalized to control isolates (*n* = 6 pairs; control isolates *n* = 6; Kruskal–Wallis test, *p < 0.05). **(B)** Western blot analysis of dagl-α and cb1 receptor. Each protein was tested concurrently with β-actin as a control. Protein expression of dominants and subordinates was normalized to communal controls as a ratio (illustrated values below each band); C, communals; D, dominants; S, subordinates. Bar graphs represent average % change in protein concentration of four replicates of samples each consisting of 10 brains normalized to WT communals.

Previously, we demonstrated that social status regulates the activation of the startle escape and swim behaviors. The sensitivity of the startle escape response significantly increases in subordinates relative to dominants and group-housed fish; while swimming frequency significantly increases in dominants and decreases in subordinates (Miller et al., 2017). Given that the escape and swim circuits receive descending and local neuromodulatory inputs, we hypothesized that the differences in excitability and behavioral selection are likely due to a rebalance in the strength of excitatory and inhibitory neuromodulatory inputs. One potential mechanism is the previously described retrograde release of 2-AG from the post-synaptic M-cell shown to regulate the M-cell’s excitability by potentiating release from pre-synaptic dopaminergic inputs (Cachope et al., 2007). Moreover, the ECS is known to modulate other brain and spinal circuits involved in regulating motivated behavior (El Manira et al., 2008; Wenzel and Cheer, 2018). This lends credence to the notion that the ECS plays a key regulatory role in the molecular mechanism by which social status impacts circuit excitability. Therefore, we measured whole brain gene expression patterns of diacylglycerol lipase (DAGL), the primary enzyme that synthesizes 2-AG, monoacylglycerol lipase (MGL), the primary enzyme to degrade 2-AG, and Cannabinoid receptor type 1 (CB_1_R) (Zou and Kumar, 2018). We found that the RNA gene expression of DAGL and CB_1_R are socially regulated (Figure 2A). Dominant animals showed a significant increase in DAGL expression relative to subordinates, and subordinates showed a significant decrease in DAGL expression relative to controls [Kruskal–Wallis test, *p* < 0.05; Figure 2A]. We also observed that the expression of CB_1_R was significantly increased in subordinates relative to controls [Kruskal–Wallis test, *p* < 0.05; Figure 2A], but CB_1_R expression was not significantly different between dominants and subordinates. Western blot analysis of DAGL and CB_1_R showed similar protein expression patterns (Figure 2B). DAGL western blots consistently showed multiple protein bands. Several causes for this are possible, including: denaturation of protein structure leading to increased antibody cross-reactivity with similar amino acid residues; differences in phosphorylation states of the DAGL proteins; and reduced antibody specificity for DAGL. Regardless, the largest bands were reliably ∼110 kDa, consistent with the size of DAGL. Such variations were not observed with CB_1_R western blots that consistently showed single bands (Figure 2B).

### 2-AG Modulation of Motor Activities Is Social Regulated

**FIGURE 3:**
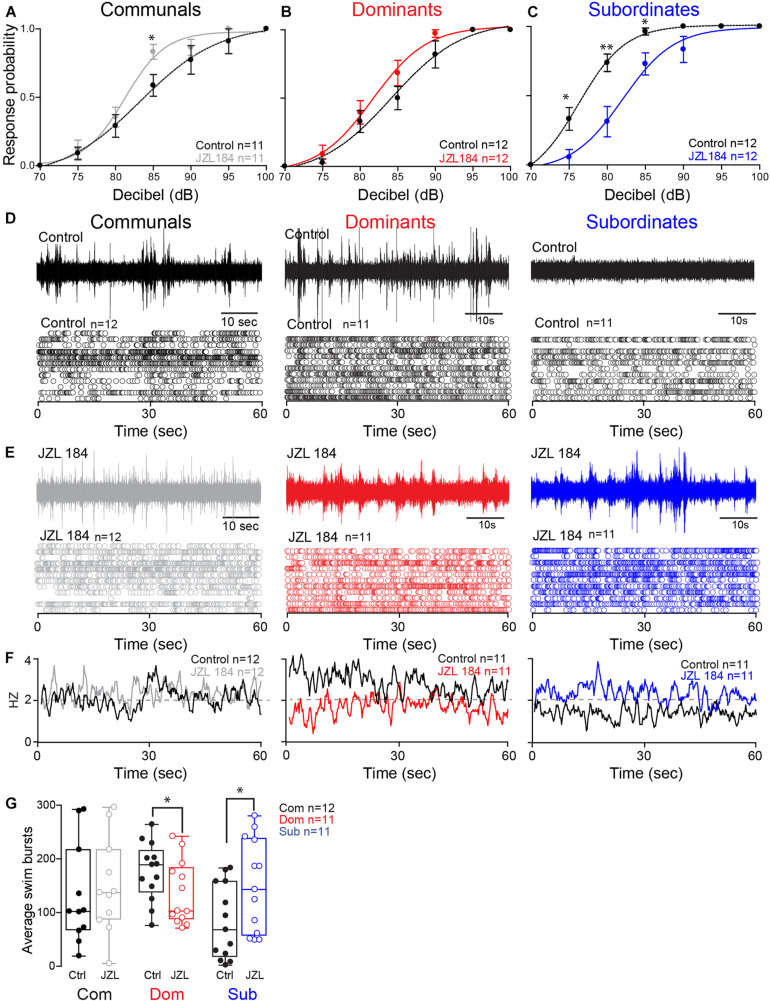
2-AG modulation of escape and swimming activities is social status-dependent. **(A–C)** Probability of startle escape response before (control) and after JZL184 injections for communals, dominants and subordinates, respectively. Asterisks (^∗^*p* < 0.05, ^∗∗^*p* < 0.005) denote statistical difference between control and experimental condition at the specified dB level. **(D)** 1 min recoding of far field-potentials of spontaneous swimming activity before (control) and **(E)** after JZL184 injections for communal, dominants and subordinates, respectively, along with respective raster plots of each condition. **(F)** Average swimming frequency for all animals tested before and after JZL184 injection. **(G)** Box and whiskers plots of the average number of swim bursts per 1 min for each social phenotype. Dots represent individual animals. The box extends from the 25th to 75th percentiles, horizontal line is the median, and whiskers represent max/min values.

These results led us to postulate that differences in the ECS may account for the social status-dependent differences in locomotor behavior. To test this hypothesis, we augmented 2-AG levels by injecting the animals with JZL184, an irreversible inhibitor of MGL (Long et al., 2009; Figures 3A–C). To compare differences in the startle escape response probabilities among the three animal groups and treatment (JZL184 injection), we performed a mixed-design ANOVA (between-subject factor as Group; within-subject factors as Treatment and Decibel). There was a significant main effect of Decibel [*F*(3.35,107.06) = 338.337, *p* < 1.0e-16; Figures 3A–C] and marginal main effect of Group [*F*(2,32) = 3.023, *p* = 6.30e-2; Figures 3A–C], but there was no effect of Treatment [*F*(1,32) = 0.080, *p* > 0.05; Figures 3A–C]. There were significant interactions of Group^∗^Treatment [*F*(2,32) = 15.69, *p* = 1.80e-5; Figures 3A–C], Group^∗^Decibel [*F*(6.69,107.06) = 2.26, *p* = 3.75e-2; Figures 3A–C], Treatment^∗^Decibel [*F*(3.75,120.06) = 2.18, *p* = 7.99e-2; Figures 3A–C], and Group^∗^Treatment^∗^Decibel [*F*(7.50,120.06) = 3.61, *p* = 1.12e-3; Figures 3A–C]. We performed the *post hoc* test to determine which animal groups had higher escape response probability. We observed that the response probability for dominants was significantly lower compared to subordinates (LSD, *p* = 3.95e-2; Figures 3A–C), but there was no difference compared to communals (LSD, *p* > 0.05; Figures 3A,B). Moreover, the response probability for subordinates was also significantly higher compared to communals (LSD, *p* = 4.41e-2; Figures 3A,C).

We then performed further analysis to determine whether JZL184 injection affected the escape response in each animal group. In communals, there was significant main effect of Decibel [*F*(3.13,31.27) = 107.87, *p* < 1.0e-16; Figure 3A], but no effect of Treatment [*F*(1,10) = 2.63, *p* > 0.05; Figure 3A]. There was also no Treatment^∗^Decibel interaction [*F*(3.47,34.72) = 1.72, *p* > 0.05; Figure 3A]. We also observed a significant difference of the startle escape responses due to JZL184 at 85 dB for communals [paired one-sample two-sided *t*-test, *t*(10) = 3.08, *p* = 1.16e-2; Figure 3A]. In dominants, there were significant main effects of Treatment [*F*(1,11) = 7.57, *p* = 1.88e-2; Figure 3B] and Decibel [*F*(2.40,26.43) = 106.12, *p* < 1.0e-16; Figure 3B]. There was no effect of Treatment^∗^Decibel interaction [*F*(2.90,31.89) = 1.43, *p* > 0.05; Figure 3B]. We did not observe a significant difference after the treatment at a particular decibel although we observed that JZL184 injection increased the overall startle escape response over the wide range of decibel. In subordinates, there were significant main effects of Treatment [*F*(1,11) = 30.08, *p* = 1.91e-4; Figure 3C], Decibel [*F*(2.15,23.65) = 134.28, *p* < 1.0e-16; Figure 3C], and Treatment^∗^Decibel [*F*(3.14,34.59) = 5.67, *p* = 8.50e-5; Figure 3C]. We observed that JZL184 injection significantly decreased the overall startle escape response over the wide range of decibels. In particular, we observed significant differences in the startle escape responses at 75 dB [paired one sample two-sided *t*-test; *t*(11) = 3.45, *p* = 5.46e-3; Figure 3C], at 80 dB [*t*(11) = 3.82, *p* = 2.86e-3; Figure 3C], and at 85 dB [*t*(11) = 2.72, *p* = 1.99e-2; Figure 3C]. In summary, the results show that blocking 2-AG degradation increased the startle escape response sensitivity in communals and moderately increased it in dominants, but significantly decreased the startle escape sensitivity in subordinates.

Activation of the startle escape response suppresses swimming activity by inhibiting the slow motor neurons that drive swimming behavior (Svoboda and Fetcho, 1996; Satou et al., 2009). Given that the ECS is implicated in promoting motivated behavior, we hypothesized that increasing the availability of 2-AG is likely to promote a behavioral switch in the activation pattern that would favor motivated behavior (i.e., swimming) over submissive behavior (i.e., escape). The notion is that augmenting 2-AG would be sufficient to reverse the activation pattern of the swim circuit in a socially dependent manner as was observed with the escape response. Indeed, we found that injection of JZL184 had the opposite effects on dominants and subordinates in that subordinates significantly increased their swimming activity while dominants significantly decreased their swimming and communal animals showed only moderate change (Figures 3D–G).

We compared the difference of swim bursts among three animal groups and treatment (JZL184 injection) and performed a mixed-design ANOVA (between-subject factor as Group; within-subject factor as Treatment). We found no main effect of Group [*F*(2,31) = 0.44, *p* > 0.05; Figures 3D–G] and no effect of Treatment [*F*(1,31) = 1.50, *p* > 0.05; Figures 3D–G]. But there was a significant effect of Group^∗^Treatment interaction [*F*(2,31) = 10.89, *p* = 2.62e-4; Figures 3D–G]. We performed further analysis to determine whether JZL184 injection affects the swim bursts in each animal group. In communals, we found a marginal main effect of Treatment [*F*(1,11) = 3.65, *p* = 8.25e-2; Figures 3D–G]. JZL184 injection slightly increased the swim bursts in communals. In dominants, there was a significant main effect of Treatment [*F*(1,10) = 6.98, *p* = 2.46e-2; Figures 3D–G]. JZL184 injection significantly decreased the swim bursts for dominants. In subordinates, there was a significant main effect of Treatment [*F*(1,10) = 9.41, *p* = 1.19e-2; Figures 3D–G]. JZL184 injection significantly increased the swim bursts for subordinates.

### Effects of AM-251 on Startle Escape and Swim Activities in Dominant and Subordinate Animals

**FIGURE 4:**
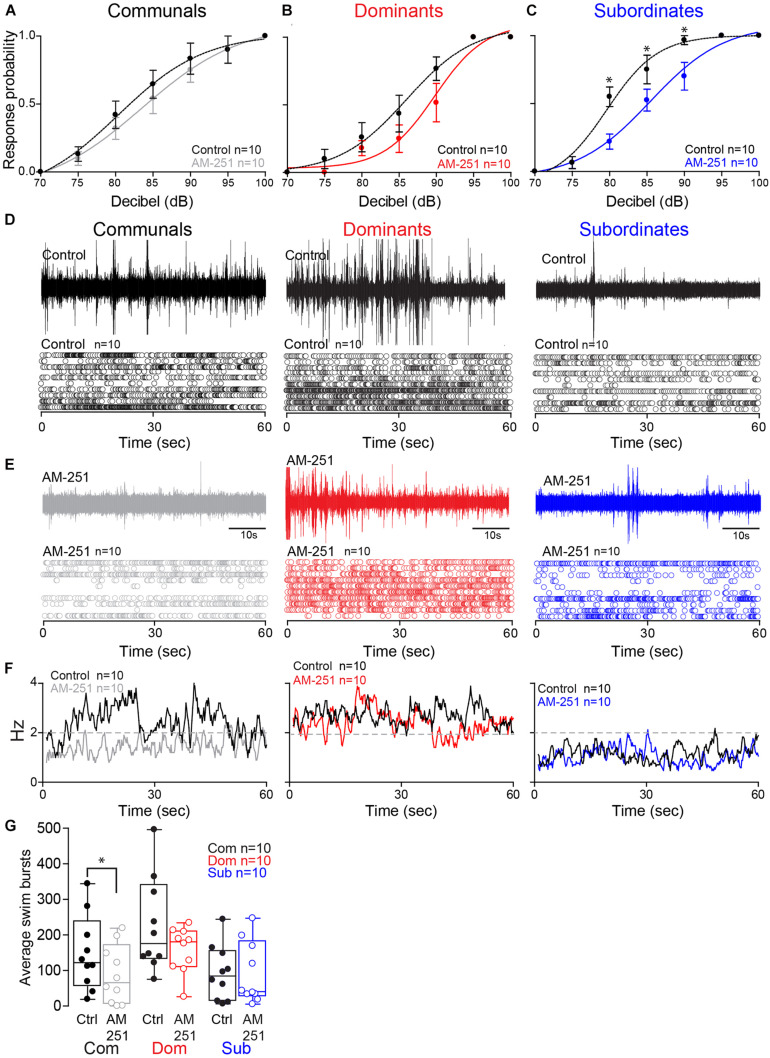
Effect of AM-251 on status-dependent escape probability and swim frequency. **(A–C)** Probability of startle escape response before (control) and after AM-251 injections for communals, dominants and subordinates, respectively. Asterisks (*p < 0.05) denote statistical difference between control and experimental condition at the specified dB level. **(D,E)** 1 min recoding of far field-potentials of spontaneous swimming activity before (control) and after AM-251 injections for communal, dominants and subordinates, respectively, along with respective raster plots for all animals tested. **(F)** Average swimming frequency for all animals tested before and after AM-251 injection. **(G)** Box and whiskers plots of the average number of swim bursts per 1 min for each social phenotype. Box plot parameters are defined in Figure 3G.

To determine whether these status-dependent differences are due to changes in CB_1_R activity, we tested the startle escape response in the presence of AM-251, a specific CB_1_R antagonist (Seely et al., 2012; Figures 4A–C). To compare the difference of the startle escape response probabilities to auditory pulses among three animal groups and treatment (AM-251 injection), we performed a mixed-design ANOVA (between-subject factor as Group; within-subject factors as Treatment and Decibel). There were significant main effects of Treatment [*F*(1,27) = 10.32, *p* = 3.39e-3; Figures 4A–C] and Decibel [*F*(2.99,80.62) = 218.56, *p* < 1.0e-16; Figures 4A–C]. But there was no main effect of Group [*F*(2,27) = 2.22, *p* > 0.05; Figures 4A–C]. We also observed significant interaction effects of Group^∗^Decibel [*F*(5.97,80.62) = 2.27, *p* = 4.55e-2; Figures 4A–C] and Treatment^∗^Decibel [*F*(2.90,78.16) = 4.36, *p* = 7.41e-3; Figures 4A–C]. But there were no effects of Group^∗^Treatment interaction [*F*(2,27) = 0.633, *p* > 0.05; Figures 4A–C] and Group^∗^Treatment^∗^Decibel interaction [*F*(5.79,78.16) = 0.76, *p* > 0.05; Figures 4A–C]. We performed the *post hoc* test to determine which animal groups had higher probability. We observed that the response probability for subordinates was marginally higher compared to dominants (LSD, *p* = 5.44e-2; Figures 4B,C). But there were no differences between subordinates and communals (LSD, *p* > 0.05; Figures 4A,C) and between dominants and communals (LSD, *p* > 0.05; Figures 4A,B).

We then performed further analysis to determine whether AM-251 injection affects the escape response in each animal group. In communals, there was significant effect of Decibel [*F*(2.70,23.30) = 46.88, *p* < 1.0e-16; Figure 4A]. But there were no effects of Treatment [*F*(1,9) = 3.32, *p* > 0.05; Figure 4A] and Treatment^∗^Decibel interaction [*F*(3.04,27.31) = 0.41, *p* > 0.05; Figure 4A]. In dominants, there was a significant main effect of Decibel [*F*(2.16,19.41) = 77.29, *p* < 1.0e-16; Figure 4B]. But there were no effects of Treatment [*F*(1,9) = 1.74, *p* > 0.05; Figure 4B] and Treatment^∗^Decibel interaction [*F*(1.74,15.66) = 1.26, *p* > 0.05; Figure 4B]. In subordinates, there were significant main effects of Treatment [*F*(1,9) = 13.50, *p* = 5.12e-3; Figure 4C] and Decibel [*F*(1.75, 15.72) = 139.13, *p* < 1.0e-16; Figure 4C]. There was also a significant effect of Treatment^∗^Decibel interaction [*F*(2.84,25.60) = 5.65, *p* = 4.63e-3; Figure 4C]. In particular, we observed significant differences in the startle escape responses at 80 dB [paired one sample two-sided *t*-test; *t*(9) = 3.69, *p* = 5.02e-3; Figure 4C], at 85 dB [*t*(9) = 2.35, *p* = 4.34e-2; Figure 4C], and at 90 dB [*t*(9) = 2.51, *p* = 3.33e-2; Figure 4C]. In summary, irrespective of social rank, AM-251 decreased startle escape sensitivity particularly so in subordinates. These findings supports Cachope et al. (2007) results in that 2-AG potentiates the sensitivity of the M-cell. We extend on their finding and show that the supply of 2-AG is socially regulated, and it modulates M-cell excitability in a status-dependent manner.

To determine whether CB_1_R activity influences swimming patterns differently among the three social groups, we compared swim bursts among three animal groups and treatment (AM-251 injection) by performing a mixed-design ANOVA (between-subject factor as Group; within-subject factor as Treatment). There were main effects of Group [*F*(2,27) = 4.25, *p* = 2.48e-2; Figures 4D–G] and Treatment [*F*(1,27) = 5.77, *p* = 2.34e-2; Figures 4D–G]. But there was no effect of Group^∗^Treatment interaction [*F*(2,27) = 1.35, *p* > 0.05; Figures 4D–G]. *Post hoc* tests showed that the swim bursts for dominants were significantly higher compared to communals (LSD, *p* = 4.61e-2; Figures 4D–G) and subordinates (LSD, *p* = 9.19e-3; Figures 4D–G). But there was no difference of swim bursts between communals and subordinates (LSD, *p* > 0.05; Figures 4D–G). We performed the further analysis to determine whether AM-251 injection affects the swim bursts in each animal group. In communals, there was a significant main effect of Treatment [*F*(1,9) = 8.05, *p* = 1.95e-2; Figures 4D–G]. AM-251 injection significantly decreased the swim bursts in communals. In dominants, there was no main effect of Treatment [*F*(1,9) = 2.48, *p* > 0.05; Figures 4D–G]. In subordinates, there was no main effect of Treatment [*F*(1,9) = 0.004, *p* > 0.05; Figures 4D–G]. Thus, blockage of CB_1_R significantly decreased swimming in communals while no changes in swimming were observed in dominants or subordinates.

Collectively, the results of supplementing 2-AG and blockage of CB_1_R indicate that 2-AG’s regulation of the swim and escape behaviors is socially regulated. More importantly, 2-AG serves as a molecular switch in shifting the activation pattern between competing circuits by modulating the balance of excitatory and inhibitory inputs onto the two motor circuits in a social status-dependent manner.

### 2-AG Regulation of M-Cell Excitability Mediated via Dopaminergic Receptor Type 1b

**FIGURE 5:**
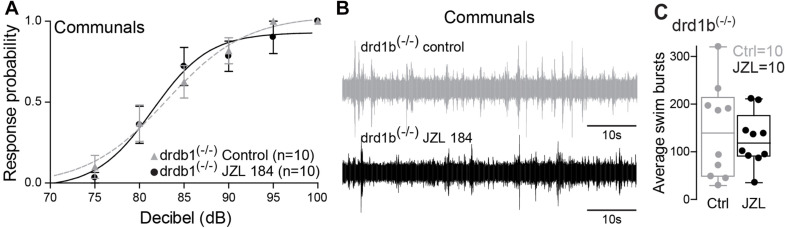
drd1b expression is socially regulated and necessary for status-dependent ECS regulation of escape and swim circuits. **(A)** Probability of startle escape response of drd1b^(–/–)^ communal zebrafish before (control) and after injection of JZL184. **(B)** 1 min recording of far field-potentials of spontaneous swimming activity before (control) and after JZL184 injections for drd1b^(–/–)^ communal zebrafish. **(C)** Average number of swim bursts per 1 min for drd1b^(–/–)^ communal fish (*n* = 10) before and after JZL184 injection.

Dopamine is known to be involved in social regulation (Watanabe and Yamamoto, 2015), motivation (Hamid et al., 2016), and aggression (Filby et al., 2010), and its function is highly interdependent with the ECS signaling pathway (Wenzel and Cheer, 2018). Specifically, ECS signaling can be mediated via dopamine receptor type 1 (DRD1) (Zenko et al., 2011). In zebrafish, the most compelling evidence is the capacity of 2-AG to potentiate mixed synaptic transmission to the M-cell that requires activation of DRD1 (Cachope et al., 2007). Moreover, evidence by Pereda et al. (1992) shows direct dopaminergic innervation of the M-cell, DA release directly potentiates M-cell excitability, and this potentiation can be blocked by antagonizing DRD1. We hypothesized that ECS signaling underlying status-dependent differences in escape sensitivity is mediated through DRD1. To test this hypothesis, we repeated our experiment of augmenting 2-AG levels but in DRD1 knockout zebrafish [drd1b^(–/–)^] (Busch-Nentwich et al., 2013). We found that startle escape sensitivity was unaffected in drd1b^(–/–)^ fish following JZL184 injection contrasting with the results of WT animals, in which an increase of 2-AG levels significantly increased startle escape sensitivity (Figure 5A compare with Figure 3A). We compared differences in the startle escape response probabilities to auditory pulses for DRD1 knockout zebrafish before and after the treatment of JZL 184 injection and performed repeated measures ANOVA (within-subject factors as Treatment and Decibel). We found a significant main effect of Decibel [*F*(2.03,18.30) = 64.03, *p* < 1.0e-16; Figure 5A]. But there was no main effect of Treatment [*F*(1,9) = 0.12, *p* > 0.05; Figure 5A] and no Treatment^∗^Decibel interaction [*F*(2.77,24.92) = 0.75, *p* > 0.05; Figure 5A]. Similarly, we found no change in swimming frequency in the drd1b^(–/–)^ animals following JZL184 injection [*F*(1,9) = 0.41, *p* > 0.05; Figures 5B,C]. These results show that the potentiating effects of 2-AG on startle escape sensitivity are mediated through DRD1b.

### Neurocomputational Analysis of 2-AG Regulation of the Escape Circuit

**FIGURE 6:**
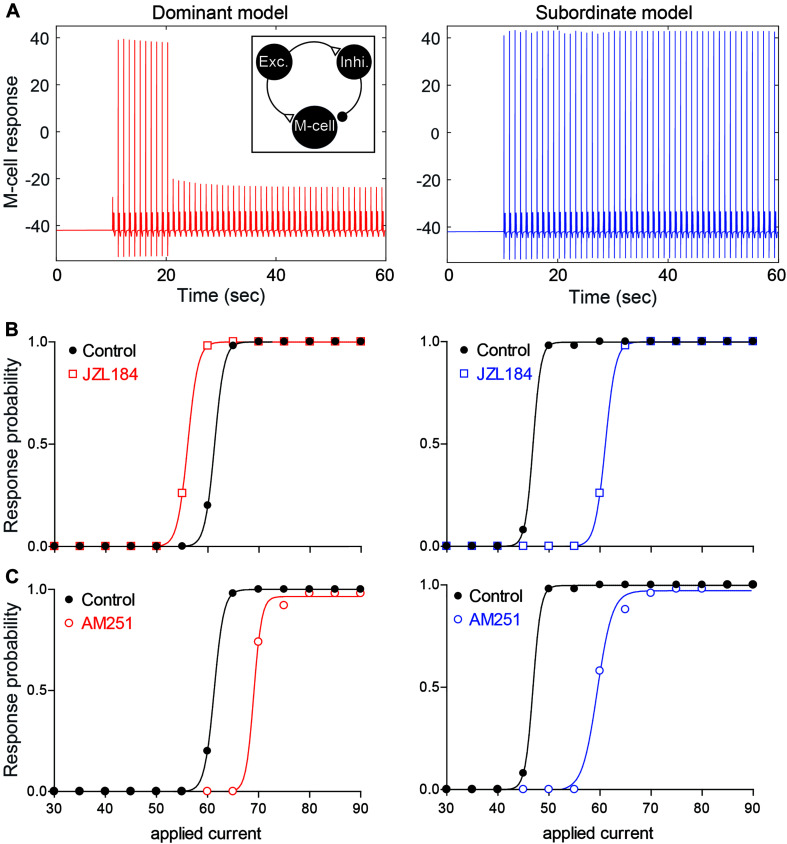
Neurocomputational models simulating the effects of 2-AG on the escape response. **(A)** Examples of the response of the M-cell model cell within a simple model network (inset) to repeated suprathreshold applied current injection in dominant-like (left) and subordinate-like (right) models. **(B,C)** Results of dominant-like and subordinate-like models simulating the probability of startle escape response before (control, black solid line) and during [JZL184, **(B)**] and blockage of CB_1_R [AM-251, **(C)**].

Our empirical results show that the ECS is affected by social experience to regulate the activation of the startle escape and swim behaviors. This complex interaction between social factors, neuromodulatory systems and motor circuits necessitated the development of a neurocomputational model to better understand how social status influences the ECS to modulate the excitability and pattern of motor activity. Toward this end, we developed a computational model whereby we simulated the M-cell along with two pre-synaptic cells, an excitatory cell (representing glutamatergic and/or dopaminergic neurons) and an inhibitory cell (representing GABAergic and/or glycinergic neurons) (see section “Materials and Methods,” Figure 6A, inset).

Using this model, we tested the hypothesis that differences in the amount of 2-AG release due to differences in DAG lipase expression would account for how 2-AG sets the gain of the pre-synaptic excitatory and inhibitory inputs onto the M-cell. More specifically, we tested whether the behavioral differences in the escape circuit are modulated by changing network properties (synaptic strengths, *g*_E→I_) and three activity levels of CB_1_Rs (CB1R_*EM*_, CB1R_*EI*_, and CB1R_*IM*_) in the model to reflect different levels of 2-AG and social status. Here, *g*_*A→B*_ and CB1R_*AB*_ represent the synaptic strength and the activity level of CB_1_R from a neuron A to a neuron B where E, I, and M represent for the excitatory, inhibitory, and M-cell, respectively. We assumed that dominants and subordinates have different levels of a synaptic strength and activity levels of CB_1_R. In particular, we assumed that low values of CB1R_*EM*_ and CB1R_*IM*_, high values of CB1R_*EI*_ and g_E→I_ for a dominant-like model while high values of CB1R_*EM*_ and CB1R_*IM*_, low values of CB1R_*EI*_ and g_E→I_ for a subordinate-like model. That is, for a dominant-like model, the inhibitory pathways were enhanced while a subordinate-like model had strong excitatory pathways.

#### Effect of Social Status on the Startle Escape Response in the Model

Depolarizing current pulses were applied to the model excitatory cell (50 stimuli for 2 ms duration with 1 sec inter stimulus interval) to determine the dynamic range of the model M-cell excitability for dominant-like and subordinate-like models. Here, the excitatory cell will excite both the inhibitory cell and the M-cell while the inhibitory cell inhibits the M-cell. In the simulation, the amplitude of the applied current, *W*_*E*_ in *I*_*app*_(*t*) on the excitatory cell, was gradually increased and the response probability of the M-cell was recorded. Figure 6A shows an example of the response of the M-cell when *W*_*E*_ = 60 for both model groups. Here, the response probabilities are 10/50 for a dominant-like model and 50/50 for a subordinate-like model. Figure 6B shows the response probabilities of the model M-cell for both model groups over the wide range of the applied currents (black curves). A subordinate-like model showed the significantly higher startle escape response probability compared to a dominant-like model. These results demonstrate that our model can reproduce the social status-dependent startle escape responses observed empirically. Different values in the synaptic strength and activation levels of CB_1_Rs were sufficient to obtain the transition of activity patterns between a dominant-like model and a subordinate-like model while maintaining the same network architecture.

#### Social Status-Dependent Effects of 2-AG on the Startle Escape Response in the Model

To determine how the social status-dependent differences of 2-AG may account for the observed changes in the startle escape circuit, we tested the excitability of the M-cell by changing the activity levels of CB_1_Rs on the presynaptic excitatory and inhibitory cells.

To mimic the observed effects of JZL184 on the startle response, we assumed that the activity levels of CB_1_Rs on all cells are increased from baseline, but in different ratios depending on the social status. Since DAGL in dominants was already higher compared to that in subordinates (Figure 2), we assumed that 2-AG level in a dominant-like model is already near the maximum capacity so that the effect of JZL184 would be negligible in a dominant-like model. On the other hand, we assumed that 2-AG level in a subordinate-like model is lower than the maximum capacity so that the effect of JZL184 would be significant in a subordinate-like model. Recall that we assumed that in dominant-like model, the activity levels of CB1R_*EM*_ and CB1R_*IM*_ are weak, but CB1R_*EI*_ is strongly active. In subordinate-like model, on the other hand, we assumed that the activity levels of CB1R_*EM*_ and CB1R_*IM*_ are strong, but CB1R_*EI*_ is weakly active. To model JZL184 injection, we used CB1R_*IM*_→1.6^∗^CB1R_*IM*_, CB1R_*EM*_→1.6^∗^CB1R_*EM*_, CB1R_*EI*_→1.4^∗^CB1R_*EI*_ for a dominant-like model and CB1R_*IM*_→1.7^∗^CB1R_*IM*_, CB1R_*EM*_→1.7^∗^CB1R_*EM*_, CB1R_*EI*_→2.7^∗^CB1R_*EI*_ for a subordinate-like model. We assumed that the activity level of CB1R_*EI*_ for a subordinate-like model would be significantly increased in the presence of JZL184. In summary, JZL184 injection in the model will enhance the activity levels of these CB_1_Rs in general. However, weakly activated CB_1_Rs will be significantly increased while strongly activated CB_1_Rs will be slightly increased although these increases of the activity levels still depend on the social status. The results show that simulated JZL184 injection slightly increased the escape response in a dominant-like model, but it significantly decreased the escape response in a subordinate-like model (compare Figures 3B,C with Figure 6B). Note that the assumption of the significant increase of the activity level of CB1R_*EI*_ for a subordinate-like model was necessary for the significant decrease of the escape response while keeping all other biophysically driven parameters constants.

To mimic the effects of the AM-251 injection on dominant-like and subordinate-like models, we assumed that the activity levels of CB_1_Rs on all cells are 0 after AM-251 injection. That is, CB1R_*EM*_ = 0, CB1R_*EI*_ = 0, and CB1R_*IM*_ = 0 for both model animal groups. These changes resulted in a slight decrease of the escape response in the dominant-like model, but a significant decrease in the subordinate-like model compared to their original response curves as we observed in the empirical results (compare Figures 4B,C with Figure 6C).

**FIGURE 7:**
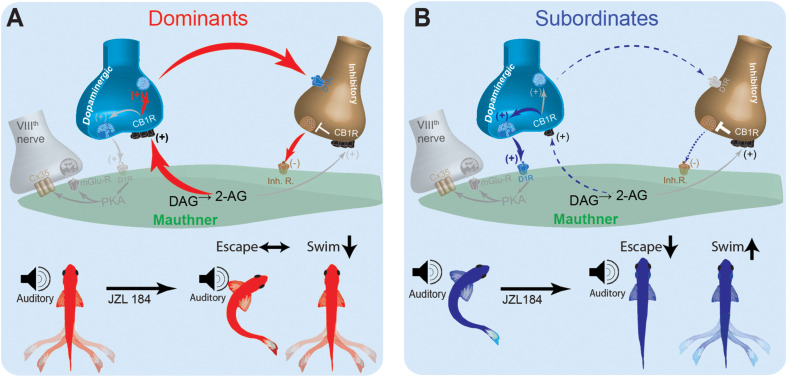
Schematic model for social status-dependent regulation of neurochemical inputs to the M-cell: The M-cell (green) receives inputs from DA cells (blue), the excitatory VIIIth cranial nerve (gray), and inhibitory (brown). Our model predicts distinct neurochemical pathways in dominants **(A)** and subordinates **(B)** responsible for differences in startle escape sensitivity. These pathways are proposed based on differential effects of JZL184 treatment on startle escape behavior (bottom). Higher baseline 2-AG in dominants is responsible for activation of the “inhibitory pathway” via inhibitory neurotransmitter release. Lower baseline 2-AG in subordinates activates the lower threshold “excitatory pathway” via the VIIIth nerve, responsible for higher startle escape sensitivity.

These results suggest that social status-dependent regulation of the pre-synaptic inputs of the M-cell may be mediated, in part, by intrinsic changes to M-cell excitability and retrograde activation of CB_1_R (Figure 7). These changes are sufficient to replicate the escape response patterns of model animal groups as observed experimentally. Model simulations suggest that in a dominant-like model, the synaptic connections in the excitatory cell → inhibitory cell → M-cell were strengthened while the connection of the excitatory cell → M-cell was weakened as compared to a subordinate-like model (Figure 7A). On the other hand, in a subordinate-like model, the synaptic connection of the excitatory cell → M-cell was strengthened while the inhibitory pathway was weakened (Figure 7B). 2-AG also differently modulates the M-cell excitability in a social status-dependent manner so that a dominant-like model has a stronger activation of CB_1_R from the excitatory cell to the inhibitory cell while a subordinate-like model has stronger activations of CB_1_Rs in the excitatory cell → M-cell, and M-cell → inhibitory cell (Figure 7).

## Discussion

Despite extensive effort to understand the neural substrates underlying the selection of context-dependent behavioral output, our knowledge remains limited of how social status affects neuromodulatory systems that regulate motor behaviors. Our study was motivated by the fact that 2-AG plays a novel role in balancing the activation between competing motor circuits, and that the ECS of vertebrates is remarkably sensitive to social influences (Song et al., 2015; Morena et al., 2016). These studies pointed to the possibility that 2-AG plays a crucial role in shifting the balance in activation of motor circuits according to social status. Here, we demonstrated that social dominance regulates the activity of the ECS to modulate the startle escape and swim motor behaviors in a behaviorally adaptive manner. We showed that JZL184 led to a partial reversal of social status-dependent motor behaviors in both dominant and subordinate zebrafish and this result provides evidence that the ECS plays a role in the neuronal modulation of social status-dependent control of the startle escape and swimming behaviors in zebrafish. Using drd1b^(–/–)^ mutants, we also showed that ECS regulation of M-cell excitability is mediated, in part, via the dopaminergic system by the activation of DRD1b. However, it should be noted that detailed future examination of the effects of drd1b mutation on social interactions and aggressive activities will be invaluable. Our behavioral observations suggest drd1b^(–/–)^ mutants pairs engaged in social agonistic interactions, formed stable dominance relationships and the time course of dominance formation mirrored that of WT animals.

One significant result of this study is the role of 2-AG in promoting synaptic transmission, a phenomenon that has been described in only a handful of studies (Cachope et al., 2007; Song et al., 2015) and is contrary to the prevalent notion that endocannabinoids suppress synaptic release (Alger, 2002; Gerdeman et al., 2002; Robbe et al., 2002; Brown et al., 2003; Chevaleyre et al., 2006; Kano et al., 2009). Our results show that prolonging 2-AG availability enhances M-cell excitability in communal animals and to a lesser extent in dominants but depresses it in subordinates. This suggests a social status-dependent dual role of the ECS within a given circuit. Studies of the ECS improved our understanding of its function in suppressing transmitter release to regulate anxiety (Moise et al., 2008), depression (Vinod and Hungund, 2006), aggression (Fontenot et al., 2018) and motor behavior (El Manira and Kyriakatos, 2010), all of which are behaviors that can be exhibited during social interactions. To our knowledge our results are novel because they demonstrate social status-dependent dual functionality of the ECS in promoting both excitation and inhibition of locomotor behavior within and across competing circuits as an adaptive strategy of social dominance.

Prolonging 2-AG availability had opposing effects on swimming activity in dominants versus subordinates. In subordinates, 2-AG increased swimming activity while in dominants swimming was decreased. How social experience influences the ECS to modulate swimming activity and the transition between escape and swimming remains unknown. A potential mechanism may lie in local control within the spinal cord motor network that modulates premotor interneurons. The fast motor neurons synthesize and release 2-AG (Song et al., 2015). It is thought that upon activation of the escape response, the fast motor neurons release 2-AG to momentarily disengage the swimming premotor elements (i.e., V2a, V1, and V0) and engage the escape circuit, thus providing a quick and local mechanism of behavioral selection (El Manira, 2014; Song et al., 2015). In this instance, the urgent execution of escape is prioritized over swimming. Although this scenario is possible in the case of subordinates, it does not explain how swimming can be selected over escape as the preferred behavior of dominants. The social regulation of swimming likely involves coordinated regulation from multiple levels of the nervous system. This is because swimming is a comparatively more flexible behavior so is likely susceptible to a greater degree of social modulation. Coordinated social regulation of swimming could take the form of chronic descending input from nuclei that control motivation coupled with spinal local control.

In addition to influencing swimming activity, prolonging 2-AG availability also induced opposite effects on the startle escape response in dominants versus subordinates. Startle escape sensitivity was significantly decreased in subordinates while it was moderately enhanced in dominants. In the startle escape circuit, it was found that 2-AG modulates the excitatory inputs to the M-cell and fast MNs (responsible for startle escape). The net effect of 2-AG is an activity-dependent potentiation of the escape circuit coinciding with a strong inhibition of the swimming circuit (Song et al., 2015). Our results for communals and dominants supported these findings although the increase of the startle escape response for dominants was moderate. This is the “clutch-like” mechanism that allows a smooth transition from swimming to startle escape, and then back to swimming (Song et al., 2015). On the other hand, our finding that 2-AG suppressed the escape circuit in subordinates is the opposite of expected effects based on the known excitatory actions of 2-AG (Song et al., 2015). Our findings suggest that, in the escape circuit, modulation of 2-AG is socially regulated, and 2-AG induces its effects by modulating presynaptic inputs onto the M-cell. Specifically, 2-AG potentiates the mixed synaptic input from the VIIIth auditory nerve onto the M-cell. The VIIIth nerve, in addition to exciting the M-cell, also excites commissural and collateral interneurons that inhibit the M-cell. M-cell firing only occurs when the direct excitatory input from the VIIIth nerve is sufficient to override the indirect inhibitory inputs (Korn and Faber, 2005).

Our results support previous findings that JZL184 treatment increases startle escape in communal zebrafish (Song et al., 2015). We found that JZL184 increased startle escape sensitivity in communals and dominants but decreased startle escape sensitivity in subordinates. Collectively, JZL184 treatment negated behavioral status-dependent differences in the startle escape response of zebrafish. This could be explained by the large increases in 2-AG concentration that ensues from inhibiting the degradative enzyme MAGL upon JZL184 administration. Previous research demonstrated that JZL184 led to a more than 5-fold increase in 2-AG levels in murine brains (Long et al., 2009). If 2-AG levels vary according to social status, then the large increase in 2-AG would eliminate any differences in 2-AG concentrations between dominants and subordinates that could be responsible for the original differences in startle escape sensitivity and explain why JZL184 administration abolished social status-dependent differences in startle escape sensitivity. Although we have no direct evidence, our results of differences in dagl expression suggest that synthesis of 2-AG is likely to differ between dominant and subordinates. Future studies quantifying 2-AG concentrations will be necessary to verify whether differences in dagl expression directly translate into differences in 2-AG availability.

Although our results of supplementing 2-AG showed clear status-dependent effects on motor activity, our results of blocking CB_1_R using AM-251 were less definitive. On one hand, our results are consistent with previous work where application of AM-251 induces a general decrease in both fictive swimming and startle escape response in communal zebrafish (Song et al., 2015). Our results along with Song et al. are in opposition to previous work in goldfish in which application of either AM-251 or SR141716 had no effect on the amplitude of the excitatory post-synaptic potential (EPSP) from the VIII^*th*^ nerve onto the M-cell (Cachope et al., 2007). These researchers reasoned that 2-AG is not released tonically from the M-cell, and so blocking the receptor would not affect the startle escape response. Our finding, that blocking CB_1_R reduces startle escape sensitivity in subordinates, suggests either of several possibilities: (1) that the systemic application of AM-251 is blocking CB_1_R upstream of the M-cell, or (2) that there is a tonic release of 2-AG in the M-cell of subordinate fish. Considering the first possibility, blocking CB_1_R on hair cells could affect the startle escape response by influencing sensitivity to sound. However, there is currently no evidence that hearing is influenced by ECS activity. The second possibility is supported by the higher startle escape sensitivity in subordinates and the known potentiating effects of 2-AG on startle escape behavior. However, this is complicated by the result that JZL184, which is reported to increase 2-AG, also reduced startle escape sensitivity in subordinates. The findings by Song et al. (2015) are hard to reconcile with our results unless these researchers unintentionally selected subordinate or chronically stressed fish for their AM-251 experiments.

In an effort to reconcile these differences and probe possible cellular mechanisms of how social status affects ECS modulation of startle behavior, we built a neurocomputational model of the escape circuit based on a simplified representation of the properties of the relevant neurons. Although our simplified model did not include all the detailed neural elements that may act *in vivo*, it enabled the reproduction of several important network activity patterns in the escape circuit observed experimentally. Neurocomputational analysis suggested that social experience induces its regulatory effects on the ECS by shifting the balance between the excitatory and inhibitory neuromodulatory pathways that control M-cell excitability (Figure 7). In dominants, enhanced expression of DAGL increases 2-AG synthesis, which promotes dopaminergic release and activation of the inhibitory inputs that in turn inhibit the M-cell (Figure 7A). This would explain why supplementing 2-AG with JZL184 had minimal effect on M-cell excitability but significantly reduced swimming frequency in dominants. Conversely, reduction in DAGL expression in subordinates reduces dopaminergic release, which reduces the strength of the inhibitory input onto the M-cell (Figure 7B). In effect, in subordinates M-cell excitability is enhanced due to removal of inhibition. This model is supported by the fact that when subordinates were injected with JZL184, their startle escape sensitivity declined while swimming activity increased; thus, shifting their motor activity from subordinate-like to dominant-like behavior. This proposed model based on empirical data was faithfully recapitulated, in part, by our neurocomputational analysis.

Note that in the neurocomputational model, we changed four parameters: the synaptic strength *g*_*E→I*_ (the synaptic strength from the excitatory cell to the inhibitory cell) and three CB_1_Rs. The differences of these four parameters between two model animal groups were about 10∼25% while network architecture was kept unchanged. Different values of CB_1_Rs in two model animal groups were essential to mimic the different effects of JZL184 injection and AM-251 injection depending on the social status. Moreover, the different values of *g*_*E→I*_ between two model groups was also essential to mimic the effect of AM-251 injection. As shown in Figure 4B, after AM-251 injections the response curve of dominants was slightly lower compared to that of subordinates. This suggests that dominants have either strong inhibitory pathways or weaker excitatory pathways onto the M-cell compared to subordinates even without the effects of CB_1_Rs. In the model, we varied values of *g*_*E→I*_ depending on the social status. One may vary other synaptic strengths (*g*_*E→M*_ or *g*_*I→M*_), but two model animal groups need to have at least one different value among three synaptic strengths to reflect different activity patterns depending on social rank.

To model the effects of JZL184, we increased the values of CB_1_R at different ratios depending on the social status and locations of CB_1_R. Some variations of these ratios qualitatively reproduced similar simulation results for both model animal groups. However, for a subordinate-like model, the incrementing ratio of CB1R_*EI*_ due to JZL184 should be significantly higher compared to those in CB1R_*EM*_ and CB1R_*IM*_ to replicate the significant decrease of the startle escape. That is, we assumed JZL184 significantly increases the excitatory → inhibitory pathways which result in the significant inhibitory input to the M-cell. Further study is needed to test our hypothesis on this pathway.

It is noteworthy to mention some limitations of the computational model. First, our model does not include all the detailed neural components that may act *in vivo*. In particular, we built a neurocomputational model for the escape circuit consisting of one M-cell, one pre-synaptic excitatory cell of the M-cell, and one pre-synaptic inhibitory cell of the M-cell to explore how social factors regulate the neuromodulatory inputs onto the M-cell. Future computational approaches may extend on the model to incorporate other feed-forward and feed-backward neuromodulatory inputs (glutamatergic, dopaminergic, GABAergic, and glycinergic neurons) known to impinge on the escape and swim circuits. These various inputs are likely to work synergistically with the ECS to differentially regulate motor circuit excitability in dominants and subordinates. The second limitation is that the model does not incorporate the swim circuit. To further explore modulation of swimming, one could combine the current model of the pre-synaptic M-cell escape circuit with a model of the swim circuit (Miller et al., 2017). However, in the absence of definitive anatomical information of the swim circuit, in particular information regarding synthesis and release of 2-AG and expression patterns of CB_1_R, results from neurocomputation models will be speculative and unlikely to provide representative insight into the ECS’s specific role in the social modulation of swimming.

In our experiments, whole brain qPCR and protein expression analysis, while informative, were not sufficient in providing tissue-specific protein expression patterns. For instance, the whole brain approach could be masking differences in CB_1_R expression patterns between dominants and subordinates that would be otherwise detected with more targeted approach. Although we have no direct evidence, we suspect that detailed expression analysis (either via IHC or *in situ* hybridization) of CB_1_R within the excitatory dopaminergic and inhibitory neurons are likely to show differences in expression patterns between the two social phenotypes. These future experiments will provide the necessary details to construct more accurate models and further our understanding of how social factors regulate network dynamics. Finally, although our study primarily focused on CB_1_R, the contribution of CB_2_R in regulating locomotor activity should not be overlooked. While CB_2_R is mostly present on immune cells and is known to regulate immune responses and inflammatory pathways (Cabral and Griffin-Thomas, 2009; Atwood and MacKie, 2010), recent evidence suggests expression and functional effects of CB_2_R in the brain in regulating anxiety-like behavior and swimming activity in zebrafish larvae (Chen et al., 2017; Acevedo-Canabal et al., 2019). Thus, future experiments examining the effects of social experience on brain expression of CB_2_R and its regulation of the startle and swim circuits will provide added insights of how social factors impinge on nervous system function and will facilitate the development of computational models that more accurately represent the structural and functional connectivity of the motor networks.

## Conclusion

We report that in zebrafish the ECS is regulated by social status to modulate the activation of startle escape and swimming in a behaviorally adaptive manner. We propose a model that this status-dependent regulation of motor activation is driven by a shift in the activation patterns of descending excitatory and inhibitory pathways that modulate the escape circuit. The report provides new insights into how social factors impact nervous system function to regulate the interactions of neuromodulatory pathways to optimize motor output.

## Data Availability Statement

The raw data supporting the conclusions of this article will be made available by the authors, without undue reservation.

## Ethics Statement

The animal study was reviewed and approved by the East Carolina University Institutional Animal Care and Use Committee. Animal Use Protocol #D320a.

## Author Contributions

SO and FI conceived and designed the experiments. SO, TM, and MK conducted the experiments. SO, TM, and FI analyzed the data. SO, SA, CP, and FI wrote and critically revised the manuscript. SA and CP developed the neurocomputational model. All authors have approved the final version of the manuscript and attested to the accuracy of the data presented.

## Conflict of Interest

The authors declare that the research was conducted in the absence of any commercial or financial relationships that could be construed as a potential conflict of interest.

## Footnotes

1 audacityteam.org

2 https://senselab.med.yale.edu/modeldb/
